# Ethical challenges in designing and conducting medicine quality surveys

**DOI:** 10.1111/tmi.12707

**Published:** 2016-05-20

**Authors:** Patricia Tabernero, Michael Parker, Raffaella Ravinetto, Souly Phanouvong, Shunmay Yeung, Freddy E. Kitutu, Phaik Yeong Cheah, Mayfong Mayxay, Philippe J. Guerin, Paul N. Newton

**Affiliations:** ^1^ Lao‐Oxford‐Mahosot Hospital‐Wellcome Trust Research UnitMahosot HospitalVientianeLao PDR; ^2^ WorldWide Antimalarial Resistance NetworkOxfordUK; ^3^ Centre for Tropical Medicine & Global HealthUniversity of OxfordOxfordUK; ^4^ The Ethox CentreUniversity of OxfordOxfordUK; ^5^ Department of Clinical SciencesInstitute of Tropical MedicineAntwerpBelgium; ^6^ Department of Pharmaceutical and Pharmacological SciencesKU LeuvenBelgium; ^7^ United States PharmacopeiaPromoting the Quality of Medicines ProgramUnited States Pharmacopeial ConventionRockvilleMDUSA; ^8^ Department of Global Health and DevelopmentFaculty of Public Health and PolicyLondon School of Hygiene and Tropical MedicineLondonUK; ^9^ Pharmacy Department and School of Public HealthMakerere University College of Health SciencesKampalaUganda; ^10^ International Maternal and Child Health UnitDepartment of Women's and Children's HealthUppsala UniversityUppsalaSweden; ^11^ Mahidol Oxford Tropical Medicine Research UnitMahidol UniversityBangkokThailand; ^12^ Faculty of Postgraduate StudiesUniversity of Health SciencesVientianeLao PDR; ^13^ Faculty of Infectious and Tropical DiseasesLondon School of Hygiene and Tropical MedicineLondonUK

**Keywords:** ethics, poor quality, medicines, surveys, falsified and substandard medicines, counterfeit, éthique, mauvaise qualité, médicaments, surveillances, médicaments falsifiés et de qualité inférieure, contrefaçon

## Abstract

**Objectives:**

In this paper we discuss the main ethical challenges related to the conduct of medicine quality surveys and make suggestions on how to address them.

**Method:**

Most evidence‐based information regarding medicine quality derives from surveys. However, existing research ethical guidelines do not provide specific guidance for medicine quality surveys. Hence, those conducting surveys are often left wondering how to judge what counts as best practice. A list of the main ethical challenges in the design and conduct of surveys is presented.

**Results and conclusions:**

It is vital that the design and conduct of medicine quality surveys uphold moral and ethical obligations and analyse the ethical implications and consequences of such work. These aspects include the impact on the local availability of and access to medicines; the confidentiality and privacy of the surveyors and the surveyed; questions as to whether outlet staff personnel should be told they are part of a survey; the need of ethical and regulatory approvals; and how the findings should be disseminated. Medicine quality surveys should ideally be conducted in partnership with the relevant national Medicine Regulatory Authorities. An international, but contextually sensitive, model of good ethical practice for such surveys is needed.

## Abstract (fr)

**Objectifs:**

Identifier et discuter les principaux enjeux éthiques liés à la conduite des surveillances et formuler des suggestions sur la façon de les aborder.

**Méthode:**

La plupart des informations fondées sur des preuves en ce qui concerne la qualité des médicaments découle des enquêtes. Cependant, les directives éthiques de recherche existantes ne fournissent pas d'indications précises pour les enquêtes sur la qualité de la médecine. Par conséquent, ceux menant des enquêtes sont souvent laissés à se demander comment juger ce qui compte comme la meilleure pratique. Une liste des principaux défis éthiques dans la conception et la conduite des enquêtes est présentée.

**Résultats et conclusions:**

Il est essentiel que la conception et la conduite des enquêtes sur la qualité de la médecine respectent les obligations morales et éthiques et analysent les implications éthiques et les conséquences d'un tel travail. Celles‐ci sont: l'impact sur la disponibilité locale et l'accès aux médicaments, la confidentialité et la protection de la vie privée des enquêteurs et des enquêtés, des questions quant à savoir si les membres du personnel de sortie doivent être informés quils font partie d'une enquête, la nécessité d'approbations réglementaires et d’éthique et la façon dont les résultats devraient être diffusés. Les enquêtes sur la qualité de la médecine devraient idéalement être menées en partenariat avec les autorités relevantes de réglementation des médicaments nationaux. Un modèle international, mais contextuellement sensible, de bonne pratique éthique pour ces enquêtes est nécessaire.

## Abstract (es)

**Objetivos:**

Identificar y discutir los principales retos éticos relacionados con la realización de encuestas y hacer sugerencias sobre la forma de abordarlas.

**Método:**

La mayor parte de la información basada en la evidencia en lo que respecta a la calidad de los medicamentos se obtiene a través de encuestas. Sin embargo, las guías éticas de investigación existentes no proveen una guía específica para las encuestas de calidad de medicamentos. Por lo tanto, aquellos que llevan a cabo las encuestas a menudo no saben cómo definir lo que se considera como “la mejor práctica”. Aquí se presenta una lista de los principales retos éticos en el diseño y en la realización de encuestas.

**Resultados y conclusiones:**

Es vital que el diseño y el pase de encuestas de calidad de medicamentos apoyen obligaciones éticas y morales, así como el análisis de las implicaciones y las consecuencias éticas de dicho trabajo. Estas son: el impacto sobre la disponibilidad local de y el acceso a las medicinas; la confidencialidad y la privacidad de los encuestados y encuestadores; preguntas sobre si los trabajadores del punto de entrega deberían ser avisados de que están formando parte de un estudio; la necesidad de aprobaciones éticas y regulatorias; y cómo diseminar los hallazgos obtenidos. Las encuestas sobre la calidad de los medicamentos deberían idealmente realizarse junto con las autoridades nacionales relevantes en regulación de medicamentos. Se requiere un modelo internacional, pero contextualmente sensible, de buenas prácticas éticas

## Introduction

Medicines are of vital importance in modern health systems, and access to quality‐assured medicines is part of the basic right to health 1, 2. Nevertheless, poor‐quality medical products, including medicines, vaccines and diagnostic devices, are widespread due to poor manufacture (substandard medicines) or deliberate falsification (falsified medicines) 3, 4, 5, 6, 7, 8, 9, 10, 11, 12, 13. These jeopardise national, regional and global attempts to improve access to effective health care because they lead to avoidable morbidity and mortality, waste financial resources, and contribute to drug resistance 6, 14, 15, 16, 17, 18, 19, 20, 21, 22. Since poor‐quality medicines result in ‘harming’ rather than ‘curing’ patients, they violate two fundamental principles of medical ethics, i.e. ‘beneficence’ and ‘non‐maleficence’ 23, 24.

Medicine quality surveys are investigations in which medicines are collected for quality testing 25, 26. They are essential to obtain evidence‐based understanding of the prevalence of substandard and falsified medicines in circulation and to design corrective actions. The reasons and ethical arguments for conducting these surveys are legitimate 23, 24 and described in Table 1. Even though quality surveys do not involve the participation of human subjects in medical experiments, they may put both the surveyors and the surveyed at risk. Furthermore, their results may inform national policies and have a direct impact on individuals and their public health. Inaccurate results due to poor methodological procedures and/or conduct (e.g. a insufficient sample size, incorrect sampling or the analysis of a partial set of samples) may lead to wrong policy recommendations, posing important ethical risks that require careful thought. It is therefore of paramount importance that all quality surveys comply with appropriate methodological and ethical standards.

**Table 1:** Ethical arguments in favour of conducting medicine quality surveys

Individuals and communities are harmed by taking medicines that are ineffective or toxic, or both. Scientifically and ethically sound surveys may help to better understand the prevalence and causes of poor‐quality medicines, and to avoid these harms in the future
There are important public health benefits to be gained from having accurate information about type of products, frequency and causes of poor‐quality medicines on the market in particular locations, so that context‐specific corrective measures can be implemented
Surveys may also provide information on banned/illegal/unregistered/unauthorised medicines which will be useful to plan how to prevent them
Provision of information to MRAs may help identify outlets who sell and/or manufacture falsified or substandard medicines in subsequent MRA/police investigations
Surveys may provide information that will facilitate technical support for improvement in the manufacturing of medicines, for example factory re‐inspections, withdrawal of importing licences, post‐marketing quality control, batch recalls, etc
The benefits to communities, health professionals and medicines purchasers of raising awareness about poor‐quality medication/medical products and how to reduce their risk, in particular by allowing their identification and corrective actions before they reach patients
Appropriate dissemination of this information to prescribers and consumers promotes the ethical principle of autonomy as it provides information facilitating medical staff and patient′s ability to determine the optimal sources of their medicines
The prevention of use of medicines containing low/insufficiently bioavailable API may prevent exposure of organisms to subtherapeutic drug levels that facilitate emergence of resistance
The preservation of already scarce financial resources that would have been spent on poor‐quality medicines
Survey findings/results may lead to improvement in government political will and commitment towards strengthening MRA's capacity to perform its roles and responsibilities
Survey results may also lead to development, revision and changes in medicines policy, especially with regard to quality assurance of medicines

Nevertheless, the existing guidelines on research ethics, such as the World Medical Association Declaration of Helsinki 27, Nuffield Council on Bioethics 28, Belmont Report 24 and the CIOMS Guidelines 23, do not provide specific guidance on the design and conduct of medicine quality surveys. Neither do most publications and policies related to the ethics of medical research 29, 30, 31, 32, 33 and to international Good Clinical Practices codes 34, 35. Thus, those conducting medicine surveys often find themselves working in contexts where ethical guidelines and legal contexts are unclear, ambiguous or non‐existent and are often left wondering whether ethical and regulatory clearance is required and what the issues are in relation to the protection of confidentiality, informed consent, etc. No consensus guidance or opinions on the ethical considerations related to medicine quality surveys have been published. The lack of national medicine regulations, human research protection mechanisms and the poor familiarity of most ethics committees with such surveys further complicate this issue.

The intentional manufacture of and trade in falsified medicines is a criminal activity. The penalties associated with such offences differ depending on the laws and regulations of the country concerned. The production and trade in substandard medicines often results from technical and human errors, or from systematic negligence, rather than fraud, but may also be considered an offence, usually against national drug laws and regulations under which the medicine regulatory authorities (MRA) operate. In some jurisdictions, the production of substandard medicines could constitute criminal negligence 36. Although criminality and negligence raise important legal challenges, healthcare providers’ failure to practice in accordance with standard of care raises important ethical questions.

As a first step to address this problem, we identify and discuss the major ethical issues related to the design and conduct of medicine quality surveys. Medicine quality surveys may be conducted by diverse groups of investigators, ranging from MRA inspectors, pharmaceutical companies, journalists, international organisations and academic researchers. All such groups should behave ethically, and consider the ethical obligations and consequences of their work, especially towards the predictable and unpredictable risks to those surveyed, those surveying, the wider public and public health.

A comprehensive ethical analysis should consider at least:

- The appropriateness and scientific soundness of the survey methodology;
- The local impact on the availability of the surveyed medicines;
- The confidentiality and privacy risks to surveyors and the surveyed;
- Whether the surveyed should be told they are part of a survey or if/when deception is acceptable;
- Which ethical and regulatory approvals should be obtained;
- How the data will be shared, who will share what, with whom and when;
- How the findings should be disseminated (e.g. should the identity of the surveyed manufactures/distributors be disclosed in reports and publications?).

There is an urgent need for more work to be carried out on the identification and analysis of these important ethical questions.

## The regulatory context

The ethical issues related to the collection of medicine samples for quality testing will inevitably depend on the context and country in which they are to be carried out as requirements for regulatory and ethical clearance vary between countries. Where possible, it is preferable to conduct medicine quality surveys in partnership with the concerned MRAs, as they are accountable to both the government and the public for the regulation and control of medical products. However, if the MRA is poorly functioning, this will not be possible 37, particularly when surveying illegal/unregistered/unlicensed outlets.

## Ethical challenges

### Impact on the local availability of and access to medicines

If investigators at small, remote outlets, sample a substantial amount of the stock of a particular medicine, the availability of such medicines may be reduced or be at risk, for example if a survey removed most antimalarials in a malarious area in a distal limb of the supply chain. Consideration should be given by the investigators to providing quality‐assured medicines in exchange for the sample after purchase. An important related concern is that health providers in the government sector may not be willing to give medicines to the survey team because their drugs have to be accurately accounted for and should only be used for the patients with evidence of having a certain disease (e.g. a positive malaria blood smear); otherwise, they may be in trouble if audited.

### Confidentiality and privacy risks to surveyors

The intentional production of and trade in falsified medicines is a criminal activity, although thankfully an unusual context for medical research. Security of survey teams may be compromised through the inappropriate disclosure of survey information. There are only a few examples in the public domain of people working on medicine quality being attacked or threatened, although such incidents may be underreported. The most extreme was the apparent attempted murder of the head of the Nigerian National Agency for Food and Drug Administration and Control, presumably related to her work 38. Therefore, the safety of mystery shoppers and overt personnel involved in surveys of medicine quality must be considered.

A mystery shopper is a person trained to visit retail stores, disguising their true identity, to collect information about the quality of service, or compliance with regulations or prices and services provided. In comparison with overt shoppers, it is thought that mystery shoppers will yield less biased information. If mystery shoppers are members of the community in which they sample, this may present particular problems if they are recognised. Care should be taken in avoiding the identification of mystery shoppers in survey reports and publications. Mystery shoppers should ensure that any information collected in the study remains confidential. A non‐disclosure agreement of identity between survey staff, their institution and the authority (such as MRAs) could be put in place.

Formal overt inspection of the outlets by MRA inspectors is likely to be the best option when the risk to mystery shoppers is thought to be too significant. Pressure on staff participating in the sampling, as well as corruption, should be taken into consideration during planning.

Safety concerns of the research team will have to be addressed if it becomes widely known that surveys are carried out by particular institutions or individuals. This is not a remote possibility – such information can be accessed on the Internet. The responsibilities of institutions and supervisors, and adequate training and support, should be discussed with the MRA, the concerned ethics committee, local authorities and the team members and their institutions; a risk assessment should be performed before the survey is undertaken.

### Whether the surveyed should be told they are part of a survey

There is currently very little information on whether medicine providers who sell poor‐quality medicines know that they are of poor quality 39, 40. Clearly, they must have a moral and ethical responsibility to ensure that what they sell is of good quality. Unfortunately, in much of the world, providers do not have the training or facilities to assess the reliability of supply channels and identify poor‐quality medicines, and they cannot rely on a stringent regulatory oversight. The risks faced by those surveyed may include:

- If overt, especially police or MRA, inspections are noted in the community and raise suspicions of the quality of medicines sold in an outlet, the outlet(s) may lose income and incur reputational risk by the community shopping elsewhere even though the results of the inspection demonstrate that the medicines supplied may be good quality.
- Outlet staff and owners selling poor‐quality medicines, but bought the drugs in good faith, may risk harm, losing income or their business and bankruptcy, perhaps being attacked or their medicines being seized by the community, as has been described 41.
- If staff in outlets selling falsified medicines inform, or are thought to have informed, MRA or research staff about those trading in such medicines, they may risk harm.
- The publication (and dissemination) of results may lead to reputational damage to specific individuals or groups (e.g. losing confidence in manufacturers from a given region or country).

For these reasons, data and samples from a survey should be coded when sent for analysis, devoid of linkage to named outlets.

### The issue of deception

There is currently no consensus as to whether it is necessary to inform outlet staff that their outlets are being sampled and which sampling approach is more appropriate 26.

On the one hand, an overt approach may allow the investigator to learn more about the samples, distribution systems for medicines and what the outlet staff know. If outlet staff are aware and anxious to avoid falsified medicines, open sampling with feedback would allow more data to be collected. It may facilitate direct improvement in the medicine supply by positively engaging with pharmaceutical retailers. If it is decided that outlet staff should be informed, the objective of the study/survey should be explained and the consent‐seeking process should take into consideration the cultural context. Bias may arise in overt surveys as providers may offer better quality medicines to give a favourable impression and avoid harming relationships with the MRA 42.

On the other hand, a mystery shopper approach increases the probability that the samples obtained will reflect what such a shopper would be sold in real life. This is clearly appropriate for MRA investigations and should also be aimed for during research surveys, as poor quality or unregistered medicines may otherwise be concealed.

A third, compromise strategy is for outlets to be sampled by mystery shoppers after being informed by the study team on a prior visit that the survey will happen at some point in the future and requesting consent for this future undisclosed visit. However, this may influence the seller's medicine selling behaviour, resulting in an inaccurate picture of the situation. Not sampling outlets that do not consent may bias the results. Further research to examine the various methods for addressing these issues and to inform the development of models of good practice is needed.

### What to do with the information once it has been collated?

There is no consensus on what should be done, how and when, for the release of public information if suspicious medicines/medical products are found 43. Clearly, public health should be the primary priority, but there remains tension between commercial interests, the need to investigate (and investigator safety) and the need to act quickly to safeguard public health.

It is clearly unethical and irresponsible for the details of the *stated* manufacturer and other sample details of poor‐quality medicines not to be reported to the MRA and the WHO Medical Product Alert System 44. There is no point in performing the survey if this is not done. Poor‐quality samples should be reported in a timely and appropriate manner to the MRA and to WHO for action, for example rapid batch recalls and public alerts. Academic research findings should be reported to MRA and the WHO Medical Product Alert, before submission for publication, as soon as the findings are considered valid and confirmed. Journals should insist in their instructions to authors that this is done. Academic research should also be made available in the public domain, for example in an open‐access peer‐reviewed scientific paper or public repository 45.

### Dissemination of findings

When there is evidence of international illicit trade in falsified medicines, the WHO Medical Product Alert System 44 and INTERPOL are able to link countries MRAs and police forces. The antimalarial quality scientific group at the WorldWide Antimalarial Resistance Network (WWARN) compiles reports of antimalarial quality globally, and they are available online through the AQ Surveyor 11, 45.

Complexities and uncertainties about the provenance of products mean that it is important for statements about samples to clearly state that the manufacturer's name and address on the product do not necessarily reflect the actual origin. Investigators should consider obtaining legal advice to ensure that samples are appropriately described in publications and reports.

The pharmaceutical companies whose ‘products’ (whether genuinely made by them or falsified by others) were found to be poor quality should be informed of the results, preferably by/with copy to the MRAs, and asked for any information on prior reports. Liaison and collaboration should be encouraged. However, the pharmaceutical industry should not make the final decision as to whether information on poor‐quality medicines is made publicly available, as they have inherent conflict of interests 36, 43. In the case of a company wishing to delay reporting, or if a company delays addressing specific requests (e.g. they could be requested to run tests on the retained samples of a given batch), a public health risk assessment should be performed urgently by a committee of key stakeholders, including independent public health and investigation experts, WHO, the relevant MRAs and the pharmaceutical company involved – with the decision based on public health and not on commercial concerns 43. The pharmaceutical industry also has the obligation to report to the MRA any product quality defects, of their own medicines, resulting in substandard products. Depending on the seriousness of the error, voluntary batch recall should be considered or, if the defect is severe, mandatory recall must take place in accordance to the product recall procedures of the MRA. Corrective actions should be implemented so that further such errors are prevented through correct implementation of Good Manufacturing Practices (GMP) and Good Distribution Practices (GDP).

Reports of poor‐quality medicines are probably more likely to be published in comparison to surveys in which medicine quality is found to be good. However, it is unethical to selectively publish bad reports over good reports as this will skew the collated data. We suggest that there is an obligation to publish all surveys whatever the results may be. Public alerts (by MRAs) should be rapid as well as reach those at most risk – guided by public health communication experts and with key messages about what to do as well as what not to do. Public engagement should be performed to reduce the risk of patients stopping taking genuine medicines and the risk that the public's loose faith in medicines or the healthcare system 2, 43.

In addition to the problems identified above, releasing information may expose the research team to legal action from the concerned company(ies), whilst not going public may result in not protecting the community from preventable harm. Such difficulties may be especially challenging if medicine sources are government or international agencies 7. This is especially a problem for distinguishing between negligence/errors in manufacturing (substandard) and degradation of pharmaceuticals in the supply chain due to poor storage – these can be hard to distinguish chemically 8, unless independent tests are run on retained samples at the manufacturer. Degradation due to poor pharmacy practice or distribution after manufacture is not the responsibility of the pharmaceutical company but substandard medicines clearly are.

These potential situations should be envisaged in the initial protocol, and strategies defined in advance, when possible in collaboration with the MRAs and other relevant national stakeholders (e.g. national malaria or tuberculosis (TB) control programs).

### The need for ethical and regulatory approval

Another unresolved question is whether medicine quality surveys should be subject to review by a research ethics committee. The WHO Standards and Operational Guidance for Ethics Review of Health‐Related Research with Human Participants includes the statement, ‘relevant authorities should ensure ethics review of health‐related research supported by an adequate legal framework’ 46. A recent publication discusses ethical review in the context of health systems research 47, 48.

We suggest that ethical scrutiny may be required if (i) the survey goes beyond MRA routine surveillance, and/or (ii) if the risk assessment shows that there is more than minimal risk to surveyors, the surveyed or the community, and/or (iii) if it is required by regulations in the country(ies) where the survey is carried out. The following steps should be considered:

- Ethical review of the survey protocol/methodology should be considered before the survey, either with preliminary discussion of the need for ethical review with the Research Ethics Committees or a formal submission in the study country(ies) and in the country of the research sponsor/coordinator 49.
- There may be circumstances in which ethical review would not be applicable, for example, if Research Ethics Committees in the study country(ies) do not exist or do not review this kind of research. Ethical review could be performed by a qualified committee elsewhere, either in the country of the research sponsor or of another research partner. The Science and Ethical Review Group (SERG) at WHO 50, 51 has developed guidelines for the establishment of scientific and ethical review bodies.
- There may be situations where ethical approval may prove difficult, depending on the restrictions of a country, for example for surveys looking at the quality of abortion pills where there are laws preventing pregnancy termination.
- Surveys should be carried out in cooperation with the concerned MRAs when possible. Should this not be appropriate 37 or possible, the decision to proceed without MRAs involvement and approval should be documented and justified.

If risk assessment suggests that there are more than minimal risks to either surveyors or the surveyed and/or if the results of the study are expected to be potentially sensitive, an *ad hoc* independent advisory board could be appointed. This could include all the pertinent skills (e.g. analytical, legal, ethical, sociological) and advise on the survey design and conduct, the communication of results and the management of any problems/incidents during and after the research. Such a board should be selected with due consideration for potential conflict of interests, for example public health should be the prime guide to decision‐making and none of the members should feel that embarrassing data on poor‐quality medicines should be suppressed.

## Conclusions

The conduct of surveys of medicine and health product quality is of great importance, particularly in settings – often in low‐ and middle‐income countries – where there is a high likelihood of the widespread use of unsafe or ineffective medicines with significant implications for public health and for the safety of individual patients. The reasons for conducting surveys are powerful, but they also present ethical challenges requiring careful thought that have had minimal discussion.

The level of risk acceptable by the survey team, the responsibilities of institutions and supervisors, and adequate training and support should be discussed with the MRA, the concerned ethics committee and local authorities if applicable, and the collection team members and their institutions; a formal risk assessment should be performed before the survey is undertaken and should be annexed to the survey protocol. Those involved in surveys should comply with appropriate methodological and ethical standards.

It is preferable to conduct medicine quality surveys in partnership with the concerned MRAs where possible. In addition, ethical clearance in the study country and collaborative partnership with local partners (such as local researchers, representative of communities, etc.) should ensure that local requirements, challenges and needs are taken in due account.

Results obtained from quality surveys may inform national policies and have a direct impact on individuals and their public health. Urgent release of information via the MRA and WHO Medical Product Alert System should be conducted for appropriate action. Public alerts should be guided by public health communication experts to avoid patients stop taking good quality medication. All these dissemination activities should be seen as morally required, for the well‐being and protection of affected communities.

Just as in other branches of medical research, scientific and methodological soundness is a fundamental prerequisite for the ethical soundness of medicine quality surveys. There needs to be much more interdisciplinary discussion of these risks to build consensus on an ethical basis for the conduct of medicine quality surveys.

## References

[1] United Nations Human Rights Council . Access to Medicines in the Context of the Right of Everyone to the Enjoyment of the Highest Attainable Standard of Physical and Mental Health: United Nations Human Rights Council, 2013 (Available from: http://ap.ohchr.org/documents/dpage_e.aspx?si=A/HRC/23/L.10/rev.1) [20th Jun 2013].

[2] Ravinetto RM , Dorlo TPC , Caudron JM , Prashanth NS . The global impact of Indian generics on access to health. Indian J Med Ethics 2013: 10: 118–120. 23697493 10.20529/IJME.2013.035

[3] Newton PN , Green MD , Fernández FM , Day NPJ , White NJ . Counterfeit anti‐infective drugs. Lancet Infect Dis 2006: 6: 602–613. 16931411 10.1016/S1473-3099(06)70581-3

[4] Caudron JM , Ford N , Henkens M , Macé C , Kiddle‐Monroe R , Pinel J . Substandard medicines in resource‐poor settings: a problem that can no longer be ignored. Trop Med Int Health 2008: 13: 1062–1072. 18631318 10.1111/j.1365-3156.2008.02106.x

[5] IOM . Countering the Problem of Falsified and Substandard Drugs. Institute of Medicine of the National Academies, 2013 (Available from: http://www.iom.edu/Reports/2013/Countering-the-Problem-of-Falsified-and-Substandard-Drugs.aspx) [13th Feb 2014]. 24872973

[6] Newton P , Green M , Mildenhall D *et al* Poor quality vital anti‐malarials in Africa – an urgent neglected public health priority. Malar J 2011: 10: 352. 22152094 10.1186/1475-2875-10-352 PMC3262771

[7] Dorlo TPC , Eggelte TA , Schoone GJ , de Vries PJ , Beijnen JH . A poor‐quality generic drug for the treatment of visceral leishmaniasis: a case report and appeal. PLoS Negl Trop Dis 2012: 6: e1544. 22666507 10.1371/journal.pntd.0001544 PMC3362618

[8] Keoluangkhot V , Green MD , Nyadong L , Fernández FM , Mayxay M , Newton PN . Impaired clinical response in a patient with uncomplicated falciparum malaria who received poor‐quality and underdosed intramuscular artemether. Am J Trop Med Hyg 2008: 78: 552–555. 18385347 PMC7610945

[9] Leslie T , Kaur H , Mohammed N , Kolaczinski K , Ord RL , Rowland M . Epidemic of *Plasmodium falciparum* malaria involving substandard antimalarial drugs, Pakistan, 2003. Emerg Infect Dis 2009: 15: 1753–1759. 19891862 10.3201/eid1511.090886 PMC2857251

[10] United States Pharmacopeial Convention (USP) . Medicines Quality Database (MQDB), 2014 (Available from: http://www.usp.org/global-health-programs/promoting-quality-medicines-pqmusaid/medicines-quality-database-mqdb [14th Apr 2014].

[11] Tabernero P , Fernandez F , Green M , Guerin P , Newton P . Mind the gaps – the epidemiology of poor‐quality anti‐malarials in the malarious world – analysis of the WorldWide Antimalarial Resistance Network database. Malar J 2014: 13: 139. 24712972 10.1186/1475-2875-13-139 PMC4021408

[12] Nishtar S . Pakistan's deadly cocktail of substandard drugs. Lancet 2012: 379: 1084–1085. 22394558 10.1016/S0140-6736(12)60277-3

[13] Ravinetto RM , Boelaert M , Jacobs J , Pouget C , Luyckx C . Poor‐quality medical products: time to address substandards, not only counterfeits. Trop Med Int Health 2012: 17: 1412–1416. 22909082 10.1111/j.1365-3156.2012.03076.x

[14] Dondorp AM , Fo Nosten , Yi P *et al* Artemisinin resistance in *Plasmodium falciparum* malaria. N Engl J Med 2009: 361: 455–467. 19641202 10.1056/NEJMoa0808859 PMC3495232

[15] White N , Pongtavornpinyo W , Maude R *et al* Hyperparasitaemia and low dosing are an important source of anti‐malarial drug resistance. Malar J 2009: 8: 253. 19906307 10.1186/1475-2875-8-253 PMC2784792

[16] World Health Organization . Substandard/Spurious/Falsely‐Labelled/Falsified/Counterfeit Medical Products. WHO, 2012 (Available from: http://apps.who.int/gb/ebwha/pdf_files/WHA65/A65_R19-en.pdf) [10 May 2012]. Report No. WHA 65.19.

[17] WHO . Substandard/Spurious/Falsely‐labelled/Falsified/Counterfeit Medical Products. Situation Report by the Secretariat, 2012 (Available from: http://apps.who.int/gb/ssffc/pdf_files/A_MSM1_INF1-en.pdf) [2 Nov 2012]. Report No. A/MSM/1/INF./1.

[18] Attaran A , Barry D , Basheer S *et al* How to achieve international action on falsified and substandard medicines. BMJ 2012: 345: e7381. 23149211 10.1136/bmj.e7381

[19] Newton PN , Amin AA , Bird C *et al* The primacy of public health considerations in defining poor quality medicines. PLoS Med 2011: 8: e1001139. 22162953 10.1371/journal.pmed.1001139 PMC3232210

[20] Pouplin T , Phuong PN , Toi PV , Nguyen Pouplin J , Farrar J . Isoniazid, pyrazinamide and rifampicin content variation in split fixed‐dose combination tablets. PLoS One 2014: 9: e102047. 25004128 10.1371/journal.pone.0102047 PMC4086978

[21] World Health Organization . Survey of the Quality of Anti‐Tuberculosis Medicines Circulating in Selected Newly Independent States of the Former Soviet Union, 2011 (Available from: http://www.who.int/tb/features_archive/surveyTBdrugeuro/en/) [10 May 2014].

[22] Laing R , Vrakking H , Fourie B . Quality and stability of TB medicines: let the buyer beware! [Editorial]. Int J Tuberc Lung Dis 2004: 8: 1043–1044. 15455587

[23] Council for International Organizations of Medical Sciences . International Ethical Guidelines for Biomedical Research Involving Human Subjects, 2002 (Available from: http://www.cioms.ch/publications/guidelines/guidelines_nov_2002_blurb.htm) [10 May 2014]. 14983848

[24] U.S. Department of Health and Human Services . The Belmont Report, 1979 (Available from: http://www.hhs.gov/ohrp/humansubjects/guidance/belmont.html) [10 May 2014].

[25] Newton PN , Lee SJ , Goodman C *et al* Guidelines for field surveys of the quality of medicines: a proposal. PLoS Med 2009: 6: e1000052. 10.1371/journal.pmed.1000052 PMC2659710 19320538

[26] Organization WH . Recommendations on the Content of a Survey Protocol for Surveys of the Quality of Medicines, 2014 (Available from: http://www.who.int/medicines/areas/quality_safety/quality_assurance/WHO_SamplingProcedures_GeneralDocument_QAS14_590_10062014.pdf?ua=1) [02 Jul 2014]. Working document QAS/14.590.

[27] The World Medical Association (WMA) . WMA Declaration of Helsinki – Ethical Principles for Medical Research Involving Human Subjects, 2013 (Available from: http://www.wma.net/en/30publications/10policies/b3/index.html) [10 May 2014].

[28] Nuffield Foundation . The Nuffield Council on Bioethics, 1991 (Available from: http://www.nuffieldbioethics.org) [24 Feb 2014].

[29] World Health Organization . Ethical Challenges in Study Design and Informed Consent for Health Research in Resource‐Poor Settings. (Available from: http://whqlibdoc.who.int/publications/2007/9789241563383_eng.pdf) [10 May 2014].

[30] Emanuel EJ , Wendler D , Killen J , Grady C . What makes clinical research in developing countries ethical? The benchmarks of ethical research. J Infect Dis 2004: 189: 930–937. 14976611 10.1086/381709

[31] Schopper D , Dawson A , Upshur R *et al* Innovations in research ethics governance in humanitarian settings. BMC Med Ethics 2015: 16: 10. 25890281 10.1186/s12910-015-0002-3 PMC4351683

[32] American Sociological Association . ASA Code of Ethics. (Available from: http://www.asanet.org/about/ethics.cfm) [25th Feb 2014].

[33] The American Anthropological Association (AAA) . Code of Ethics of the American Anthropological Association, 1998 (Available from: http://www.aaanet.org/committees/ethics/ethicscode.pdf) [25th Feb 2014].

[34] ICH . Guideline for Good Clinical Practice, 1996 (Available from: http://www.ich.org/products/guidelines/efficacy/article/efficacy-guidelines.html) [24 March 2014].

[35] World Health Organization . Guidelines for Good Clinical Practice (GCP) for Trials on Pharmaceutical Products, 1995 (Available from: http://apps.who.int/medicinedocs/pdf/whozip13e/whozip13e.pdf) [24 March 2014].

[36] Cockburn R , Newton PN , Agyarko EK , Akunyili D , White NJ . The global threat of counterfeit drugs: why industry and governments must communicate the dangers. PLoS Med 2005: 2: e100. 15755195 10.1371/journal.pmed.0020100 PMC1062889

[37] Francis PA . The Tip of an ICEBERG, 2003 (Available from: http://www.pharmabiz.com/article/detnews.asp?articleid=15677&sectionid=47:Pharmabiz.com) [24 March 2014].

[38] Aldhous P . Counterfeit pharmaceuticals: murder by medicine. Nature 2005: 434: 132–136. 15758966 10.1038/434132a

[39] Syhakhang L , Freudenthal S , Tomson G , Wahlstrom R . Knowledge and perceptions of drug quality among drug sellers and consumers in Lao PDR. Health Policy Plan 2004: 19: 391–401. 15459164 10.1093/heapol/czh054

[40] Khan M , Akazawa M , Dararath E *et al* Perceptions and practices of pharmaceutical wholesalers surrounding counterfeit medicines in a developing country: a baseline survey. BMC Health Serv Res 2011: 11: 306. 22074046 10.1186/1472-6963-11-306 PMC3225320

[41] Newton PN , McGready R , Fernandez F *et al* Manslaughter by fake artesunate in Asia – will africa be next? PLoS Med 2006: 3: e197. 16752952 10.1371/journal.pmed.0030197 PMC1475657

[42] Madden JM , Quick JD , Ross‐Degnan D , Kafle KK . Undercover careseekers: simulated clients in the study of health provider behavior in developing countries. Soc Sci Med 1997: 45: 1465–1482. 9351137 10.1016/s0277-9536(97)00076-2

[43] Newton PN , Tabernero P , Dwivedi P *et al* Falsified medicines in Africa: all talk, no action. Lancet Glob Health 2014: 2: e509–e510. 25304415 10.1016/S2214-109X(14)70279-7

[44] WHO . Medical Product Alert. (Available from: http://www.who.int/medicines/publications/drugalerts/en/) [24 March 2014].

[45] Tabernero P , Newton PN . The WWARN antimalarial quality surveyor. Pathog Glob Health 2012: 106: 77–78. 22943540 10.1179/204777312X13419245939520 PMC3411114

[46] WHO . Standards and Operational Guidance for Ethics Review of Health‐Related Research with Human Participants, 2011 (Available from: http://whqlibdoc.who.int/publications/2011/9789241502948_eng.pdf) [24 March 2014]. 26269877

[47] Hyder AA , Rattani A . Changing the discourse on health systems research: response to open peer commentaries on “ethical review of health systems research in low‐ and middle‐income countries: a conceptual exploration”. Am J Bioeth 2014: 14: W1–W2. 24521342 10.1080/15265161.2014.881212

[48] Hyder AA , Rattani A , Krubiner C , Bachani AM , Tran NT . Ethical review of health systems research in low‐ and middle‐income countries: a conceptual exploration. Am J Bioeth 2014: 14: 28–37. 24521334 10.1080/15265161.2013.868950

[49] Ravinetto R , Buvé A , Halidou T *et al* Double ethical review of North‐South collaborative clinical research: hidden paternalism or real partnership? Trop Med Int Health 2011: 16: 527–530. 21410849 10.1111/j.1365-3156.2011.02732.x

[50] WHO . Ethical Issues in Patient Safety Research: Interpreting Existing Guidance. (Available from: http://apps.who.int/iris/bitstream/10665/85371/1/9789241505475_eng.pdf: 2013 978 92 4 150547 5) [24 March 2014].

[51] WHO . Quality Practices in Basic Biomedical Research. (Available from: http://www.who.int/tdr/publications/documents/quality_practices.pdf) [24 March 2014].

